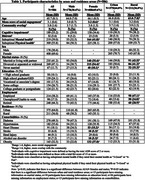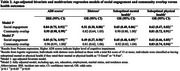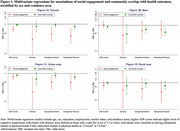# The Associations of Social Engagement with Cognitive and Physical Health in American Indian and Alaska Native Adults

**DOI:** 10.1002/alz70860_106317

**Published:** 2025-12-23

**Authors:** Jiahui Dai, Andrew Thais, Yuxi Shi, Wenjun Fan, Erin M. Poole, Spero Manson, Luohua Jiang

**Affiliations:** ^1^ Joe C. Wen School of Population & Public Health, Henry and Susan Samueli College of Health Sciences, University of California, Irvine, Irvine, CA, USA; ^2^ University of Pennsylvania, Philadelphia, PA, USA; ^3^ University of California Irvine, Irvine, CA, USA; ^4^ University of Colorado Anschutz Medical Campus, Aurora, CO, USA

## Abstract

**Background:**

Research on social engagement, community connectedness, and health outcomes in American Indian and Alaska Native (AI/AN) adults is limited. This study examined associations of social engagement and community overlap with cognitive function, distress, and mental and physical health in middle‐aged and older AI/AN adults.

**Method:**

Data were collected from a cross‐sectional survey conducted from 2019‐2023 among urban and rural AI/AN volunteers aged 45+ years residing in the Rocky Mountain area. Social engagement was measured using a five‐item social frequency instrument, and community overlap was assessed using the Inclusion of Community in Self scale. Health outcomes included the number of “Yes” responses to culturally tailored Ascertain Dementia 8‐item Questionnaire (i.e., AD8 scores), distress measured by the Kessler Psychological Distress Scale (K6), and self‐reported mental and physical health. Mental and physical health assessments were based on a four‐point Likert scale (‘1‐Excellent’, ‘2‐Very good’, ‘3‐Good’, ‘4‐Fair’), with ratings of ‘3‐Good’ or ‘4‐Fair’ classified as suboptimal. Poisson regression was applied to model associations of social engagement and community overlap with AD8 scores, while logistic regression was used to assess their associations with the other outcomes.

**Result:**

The study included 586 AI/AN participants, of whom 69.6% were female and 64.0% resided in urban areas (Table 1). Greater social engagement and community overlap were significantly associated with reduced risks of adverse cognitive and physical health outcomes (Table 2). In multivariate models, greater social engagement was significantly associated with lower AD8 scores [incidence rate ratio (IRR)=0.76], lower odds of distress [odds ratio (OR)=0.44], suboptimal mental health (OR=0.59), and suboptimal physical health (OR=0.52) among AI/AN females but not males. Conversely, among AI/AN males, greater community overlap was significantly or marginally associated with lower AD8 scores and reduced odds of the other outcomes. Urban AI/AN adults exhibited stronger associations than those in rural areas (Figure 1).

**Conclusion:**

Higher levels of social engagement and community overlap were associated with better cognitive and physical outcomes among these AI/AN volunteers, emphasizing the need for culturally tailored health interventions to strengthen social connections and enhance well‐being of AI/AN adults. Future investigations on AI/AN sex and residence differences in these relationships are warranted.